# Protocol for transcutaneous tumor photolabeling to track immune cells *in vivo* using Kaede mice

**DOI:** 10.1016/j.xpro.2024.102956

**Published:** 2024-03-20

**Authors:** Isaac Dean, Bethany C. Kennedy, Zhi Li, Fedor Berditchevski, David R. Withers

**Affiliations:** 1 Institute of Immunology and Immunotherapy, The University of Birmingham, B15 2TT Birmingham, UK; 2 Division of Radiotherapy and Imaging, The Institute of Cancer Research, SW3 6JB London, UK; 3 Institute of Cancer and Genomics, The University of Birmingham, B15 2 TT Birmingham, UK

**Keywords:** Cell Biology, Cell culture, Cell isolation, Single Cell, Flow Cytometry, Cancer, Immunology, Model Organisms

## Abstract

Preclinical tumor models have advanced our understanding of the tumor microenvironment. However, the temporal dynamics of cellular recruitment and retention within these models is poorly understood. Here, we present a protocol using transcutaneous labeling of the tumor compartment using subcutaneous and orthotopic tumors. We describe the process of cell line implantation and photoconversion of tumors to differentiate newly recruited cells from those retained within tumors. Photoconversion enables tracking of both immune cell recruitment to tumors and egress to the lymphatics.

For complete details on the use and execution of this protocol, please refer to Li et al.^1^ and Molostvov et al.^2^

## Highlights

• Engraftment of both subcutaneous and mammary fat pad preclinical tumor models
• *In vivo* transcutaneous labeling of intratumoral immune cells using near-UV light
• Detection of defined immune cell types and identification of migratory behavior

## Abstract (editor highlights)

Publisher’s note: Undertaking any experimental protocol requires adherence to local institutional guidelines for laboratory safety and ethics.

## Graphical abstract

**Figure:**
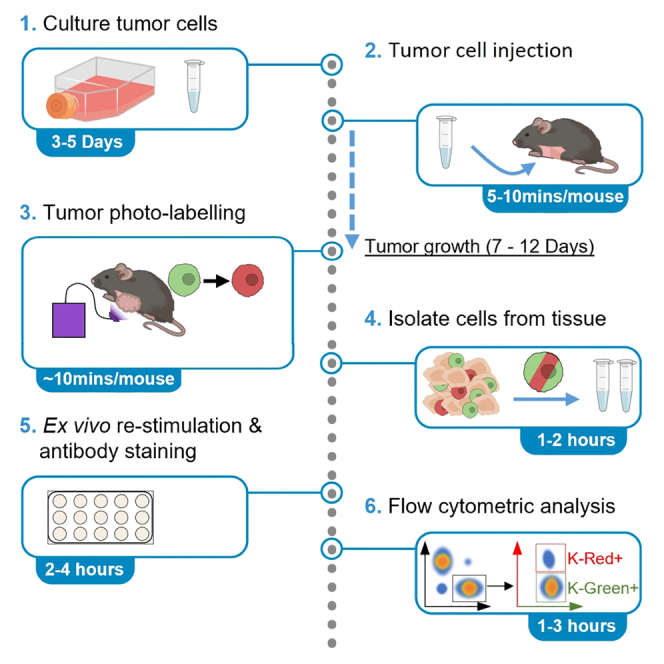

## Before you begin

This protocol describes the establishment of subcutaneous (s.c.) tumors using the colorectal cell line MC38, and orthotopic breast tumors using E0771 cells. Both subcutaneous and mammary fat pad (m.f.p) tumors provide an easily accessible tumor that can be readily exposed transcutaneously to the near-UV light required for Kaede photoconversion. Whilst in this paper we describe two cell lines, in practice this approach is applicable to any non-pigmented tumor cell lines. The main limitation of photoconversion is light penetration, therefore tumor size, pigmentation (such as melanin produced by B16F10 melanoma cells), and depth of tumor implantation must be considered when attempting transcutaneous photolabeling.

This protocol can be divided into three sections: culture and inoculation of tumor cells, Kaede photoconversion using near-UV light, and the isolation and acquisition of intratumoral cells.

### Institutional permissions

All animal procedures were approved by the University of Birmingham Welfare and Ethical Review Body in accordance with Home Office Guidelines under a Project License awarded to D.R. Withers. Kaede mice (C57BL/6 background) were obtained under MTA from the RIKEN BRC Experimental Animal Division (https://mus.brc.riken.jp/en/). All mice were bred and maintained at the Biomedical Services Unit at the University of Birmingham.

### Cell lines

MC38 adenocarcinoma cells were kindly provided by Dr. Gregory Sonnenberg (Weill Cornell Medicine, New York, NY), and E0771 mammary carcinoma cells were purchased from CH3 BioSystems (Amherst, NY). E0771^pLVx^ and E0771^Tspan6^ cells were developed by transducing parental cells (E0771) with the control lentivirus (pLVx-IRES-Puro) or lentivirus encoding Tspan6. Lentiviral transduction of E0771 is not critical to this protocol. Cells were grown in log phase in RPMI (MC38) or DMEM (E0771), further supplemented with 2 mM L-glutamine (#21875034; Thermo Fisher Scientific), 10% FBS (#F9665; Sigma-Aldrich), and 100 U/ml Penicillin/ 100 μg/mL Streptomycin (#15140122; Gibco) at 37°C and 5% CO_2_.

### Panel design

**Timing: 1–2 h**

**CRITICAL:** Combining multiple fluorescent proteins (Kaede green and Kaede red), with a panel of fluorochromes requires careful consideration of the combinations to ensure high-quality analysis. Here we show an optimized fluorochrome combination using a BD LSR Fortessa X-20 flow cytometry. Given the spectral similarities of Kaede green to FITC, and Kaede red to PE/PE-Texas Red, analysis of Kaede green/red should be possible on all flow cytometers with similar technical specifications.

1. BV510 causes a large spectral spread of Kaede green signal. It is recommended to use BV510 only if targeting a highly expressed antigen.
2. Both Kaede red and PE-Dazzle594 are excited by 488 nm and 561 nm lasers requiring considerable compensation between the two signals.
3. Due to similar emissions, combining Kaede red, PE-Dazzle594, and BV605 requires consideration to ensure populations are easily distinguished, it is therefore recommended antigens stained for with PE-Dazzle594 and BV605 should have differential expression.

Where possible, it is recommended to not use BV510, and to use either BV605 or PE-Dazzle594.

## Key resources table

**Table:** 

REAGENT or RESOURCE	SOURCE	IDENTIFIER
**Antibodies (dilution)**		

Rat anti-mouse CD16/CD32 (1:200)	BD Biosciences	#553142; RRID: AB_394656
Rat anti-mouse CD45, clone 30-F11 (1:200)	BioLegend	#103139; RRID: AB_2562341
Hamster anti-mouse CD3e, clone 145-2C11 (1:200)	BD Biosciences	#612771; RRID: AB_2870100
Rat anti-mouse CD4, clone RM4-5 (1:200)	BioLegend	#100551; RRID: AB_11218992
Rat anti-mouse CD8a, clone 53–6.7 (1:200)	BioLegend	#100721; RRID: AB_312760
Rat anti-mouse Foxp3, clone FJK16s (1:100)	Thermo Fisher Scientific	#48-5773; RRID: AB_1518812
Rat anti-mouse CD11c, clone N418 (1:300)	BioLegend	#117347; RRID: AB_2563654
Rat anti-mouse MHCII, clone M5/114.15.2 (1:400)	BioLegend	#107635; RRID: AB_2561397
Rat anti-mouse F4/80, clone BM8 (1:200)	Thermo Fisher Scientific	#50-4801-82; RRID:AB_11149361
Rat anti-mouse LY-6C, clone HK1.4 (1:300)	BioLegend	#128037; RRID: AB_2562630
Rat anti-mouse LY-6G, clone 1A8 (1:300)	BioLegend	#108441; RRID: AB_2562401
Rat anti-mouse B220, clone RA3-6B2 (1:200)	BD Biosciences	#563793; RRID: AB_2738427

**Chemicals, peptides, and recombinant proteins**		

LIVE/DEAD Fixable Near-IR Dead Cell Stain Kit (1:1,000 dilution)	Thermo Fisher Scientific	#L10119
BD Cytofix/Cytoperm Fixation/Permeabilization Kit	BD Biosciences	#554714
eBioscience Foxp3/Transcription factor staining buffer set	Thermo Fisher Scientific	#00-5523-00
TrypLE Express Enzyme (1X), phenol red (referred to as TrypLE Express)	Thermo Fisher Scientific	#12605010
Collagenase D	Roche	#11088866001
DNase I	Roche	#10104159001

**Experimental models: Cell lines**		

E0771	CH3 BioSystems	
E0771^pLVx^	Molostvov et al.^2^	
E0771^Tspan6^	Molostvov et al.^2^	
MC38	Li et al.^1^	

**Experimental models: Organisms/strains**		

B6.Cg-Tg(CAG-tdKaede)15Utr	RIKEN	(https://mus.brc.riken.jp/en/)

**Software and algorithms**		

FlowJo	BD Biosciences	https://www.flowjo.com/solutions/flowjo

**Other**		

Dymax BlueWave QX4 fitted with a VisiCure (405 nm) LED head and 8 mm focusing lens	Dymax	https://dymax.com/products/equipment/light-curing-equipment/spot-curing-systems/bluewave-qx4

## Materials and equipment

### Mice

*In vivo* tracking of cell populations requires use of transgenic animals expressing a bright photoconvertible protein such as Kaede,^3^ KikGR,^4^ H2B-Dendra2.^5^

**Figure 1:**
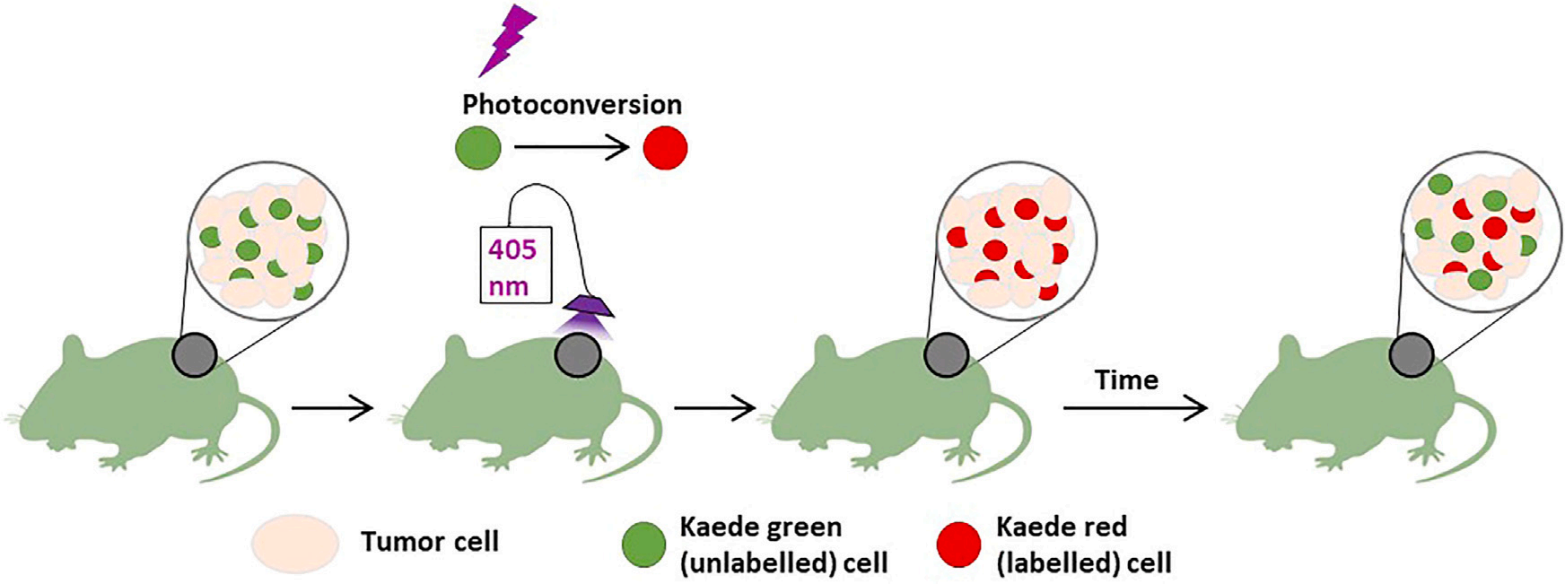
Cartoon depicting photoconversion of a subcutaneous tumor on the flank Representation of tumor photoconversion using 405 nm light to convert the native kaede green+ host cells to the kaede red version. After time, newly infiltrating hosts cells are distinguished from those retained by the presence of kaede green, and lack of kaede red protein within the cells. Kaede green and/or red can be detected by flow cytometry or imaging, and visually identified due to different emission wavelengths (518 nm kaede green and 580 nm for kaede red).

While we haven’t tested photoconversion of KikGR, previously we have validated the use of Kaede and H2B-Dendra2 mice to label peripheral lymph nodes.^6^ In our experience the signal from kaede is brighter and easier to detect than Dendra, prompting the exclusive use of Kaede mice to photo-label subcutaneous and breast tumors.^1^ Photoconvertible proteins emit light in their native state, and upon exposure to protein specific wavelengths of light (350–405 nm for Kaede, KikGR, and Dendra2), undergo changes resulting in emission of light with longer wavelengths. The Kaede tetramer undergoes photo-switching from 518 nm (Kaede green) fluorescence in its native state, to 580 nm in the converted Kaede red form when exposed to UV light (350–405 nm) (Figure 1).^7^ 6 – 8-week-old female Kaede mice were used for all *in vivo* trafficking experiments.

### Reagents

#### Culture media

DMEM or RPMI supplemented with 10% FCS, 2 nm L-glutamine, 100 U/ml Penicillin, and 100 μg/mL Streptomycin. Store at 4°C ready for use for up to 2 weeks.

#### FACS buffer

PBS supplemented with 2.5 mM EDTA and 2% FCS. Store at 4°C ready for use for up to 4 weeks.

**Table:** Gey’s Solution

Solution	Reagent	Amount (g unless stated otherwise)	Final stock volume (mL)
A	NH_4_Cl	35	500
	Gelatin	25	
	Glucose	5	
	KCl	1.85	
	KH_2_PO_4_	1.5	
	1% Phenol Red	1.5 mL	
B	MgCl_2_.6H_2_O	4.2	1000
	CaCl_2_	3.5	
	HgSO_4_.7H_2_O	1.4	
C	NaHCO_5_	22.5	1000

Gey’s red blood cell lysis solution is made from 10 mL Solution A, 2.5 mL Solution B, 2.5 mL Solution C, and 35 mL distilled water. The final solution should be stored at 4°C ready for use for up to 4 weeks.

#### Enzyme digestion mix

RPMI supplemented with 1 mg/mL Collagenase D and 0.1 mg/mL DNase I.

### Flow cytometer – BD LSR Fortessa X-20

Data were acquired on a BD LSR2 Fortessa X-20 flow cytometer with the following configuration. Dyes requiring careful consideration are marked with an asterisk.

**Table:** BD LSR Fortessa X-20 Configuration

Laser	Filter	Dye(s) tested	Voltage
355 nm UV	740/35	BUV737	720
	379/28	BUV395	620
405 nm Violet	450/50	BV421	420
	525/50	BV510∗ (Not advised)	400
	610/20	BV605∗	550
	670/30	BV650	650
	710/50	BV711	500
	780/60	BV786	620
488 nm Blue	530/30	Kaede green	320
	670/30	PerCP-Cy5.5 (Not tested)	N/A
561 nm Yellow/Green	586/15	Kaede red	480
	610/20	PE-Dazzle594∗ (Not advised)	400
	670/30	PE-Cy5 (Not tested)	N/A
	710/50	PE-Cy5.5 (Not tested)	N/A
	795/71	PE-Cy7	475
640 nm Red	671/30	APC	550
	722/44	AF700	500
	795/71	LIVE/DEAD Near-IR	500

***Note:*** Other flow cytometers and fluorochrome formats are readily available, different combinations should be tested to ensure compatibility with Kaede green and Kaede red, especially due to 488 nm and 561 nm cross excitation of the Kaede red protein.

## Step-by-step method details

### Preparation of tumor cells

**Timing: ∼1 week**

MC38 tumor cells were collected for subcutaneous injection, and E0771 cells for mammary fat pad injection.

1. Culture cells in an appropriate culture media at 37°C with 5% CO_2_ until reaching ∼70–80% confluency within T75 culture flasks.

***Note:*** MC38 cells were culture in RPMI based media, and E0771 in DMEM due to cell line preference.

2. Prewarm an appropriate culture media 37°C and bring TrypLE Express and PBS up to room temperature.

***Note:*** Alternative trypsin equivalent reagents may require warming to 37°C.

3. Remove the media from the culture flasks and gently wash the cells with PBS.
4. Add 3 mL TrypLE Express and place the flask back into the incubator for 2–3 min. Check to ensure cells have detached, gentle shake flask if required.

**CRITICAL:** Optimize digestion time for each specific trypsin-like reagent to prevent digestion influencing cellular viability.

5. Add 7 mL culture medium to the culture flasks, gently resuspend cells, and add to a 50 mL centrifuge tube.
6. Centrifuge cells for 5 min at 300–400 × *g* at 4°C.
7. After resuspending the cell pellet in 10 mL PBS, take 10 μL out to calculate cell concentration with either a manual or automatic hemocytometer, then centrifuge the 50 mL tube again as before.
8. Resuspend the cells in the appropriate volume of PBS to obtain 2.5 x 10^6^/mL MC38 and/or 5 x 10^6^/mL E0771 cells.
9. Transfer 1 mL of either 2.5 x 10^6^/mL (MC38) or 5 x 10^6^/mL (E0771) cells into 1.5 mL Eppendorf tubes ready for injections, and store on ice.

**CRITICAL:** Keep cells on ice and inject them into mice within an hour to maintain cell viability.

### Tumor cell line injection

**Timing: ∼2 weeks**

MC38 tumor cells were collected for subcutaneous injection, and E0771 cells for mammary fat pad injection.

10. Ensure all instruments have been sterilized and wipe down all surfaces with 70% ethanol.
11. Prepare mice for injection.
  a. Anesthetize mice with 5% isoflurane in an induction chamber.
  b. Transfer the mouse into the nose cone, check the depth of anesthesia by testing the pedal reflex, and maintain a depth of anesthesia using ∼2% isoflurane.
    ***Note:*** Continuously monitor anesthetized mice to ensure a steady level of anesthesia and adjust the concentration of isoflurane where necessary.
  c. For s.c. shave the flank, or for m.f.p injection shave around the 3^rd^ nipple.
    ***Note:*** Injection into the 4^th^ or 5^th^ nipple is more commonly done and is compatible with this protocol.
  d. Clean shaven area with either Hibiclens, 70% ethanol, or other appropriate disinfectant.
12. Tumor cell injection.
  a. S.c. injection; gently insert a sterile needle into the flank and inject 100 μL of 2.5 x 10^6^/mL MC38, a successful injection will cause a skin bulge at the inoculation site.
  b. M.f.p injection; using sterile forceps raise the nipple and gently insert a sterile needle through the base of the nipple before injecting 50 μL of 5 x 10^6^/mL E0771 cells into the fat pad. Successful injection will give some resistance to injection and will form a palpable ball within the fat pad.
    **CRITICAL:** Raising the nipple allows the injection to be almost parallel to the skin minimizing the risk of intraperitoneal injection.
  c. Clean the injection site with Hibiclens or other appropriate disinfectant and allow the mouse to recover from the effects of anesthesia.
  d. Provide additional support through fluids or analgesia where required.
    ***Note:*** The ideal tumor size for photoconversion is approximately 6 mm in diameter using this protocol. Both MC38 and E0771 tumors should form a tumor of this size after 12 days of tumor growth.

#### *In vivo* photo-labeling

**Timing: ∼10 min per animal**

**Figure 2:**
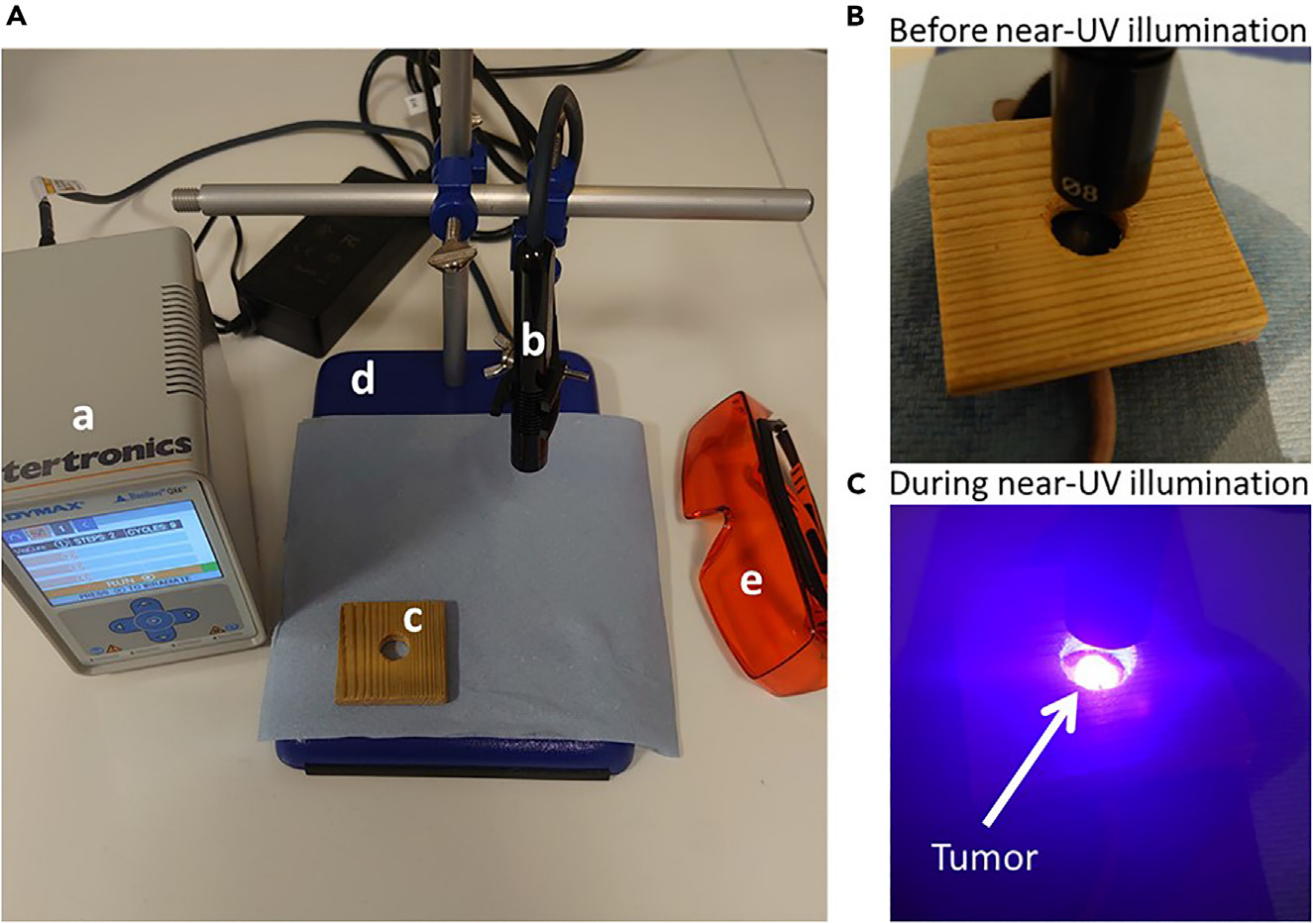
Equipment setup and procedure of tumor photoconversion After sterilizing the work area, prepare the following instrumental setup: (a) Dymax BlueWave QX4, (b) VisiCure (405 nm) LED with 8 mm lens, (c) light shield, (d) clamp stand, and (e) UV eye protection (A). Not included by required: Isoflurane nebulizer and isoflurane induction chamber. Images showing procedure before (B) and during (C) the photoconversion procedure. Example of subcutaneous tumor photoconversion is shown.
  b. Holding the LED lightguide approximately 10 mm from the tumor, turn on the Dymax BlueWave QX4 system exposing the tumor to light.
  c. In the 0% intensity steps in each cycle, carefully apply H_2_O to the tumor to prevent the skin drying out and cracking during the procedure.
  d. After the nine cycles of light exposure allow the mouse to recover from effects of anesthesia.
    ***Note:*** Whilst this protocol recommends the use of nine light exposure cycles, photoconversion can be achieved using different cycle counts with a similar total exposure time. Given the concern of skin drying out and/or becoming damaged, it is recommended to use at least four cycles of intermittent light exposure and optimization of both the number of exposure cycles, and the exposure time per cycle.
16. Aftercare.
  a. Place mice over a heated mat or within a heated chamber to aid recovery from anesthesia, and from potential temperature loss due to administering water between light exposure cycles.
  b. Provide additional support through fluids or analgesia where required.

***Note:*** It is common and expected that immediately after photoconversion the area of skin exposed appears red in color, returning to normal by the following day (within 24 h). Prolonged or excessive exposure to near UV light can damage the skin. In rare cases dry, burnt, or damaged skin can progress to tumor ulceration. Other side effects including changes in animal behavior, hair loss, or other notable sickness have not been observed during/after photoconversion, but these should be considered if animals are undergoing additional procedures in addition to light exposure.

13. Set up the Dymax BlueWave QX4 wave light curing system.
  a. Plug in all cables ensuring the VisiCure (405 nm) LED head is fitted with the 8 mm focusing lens.
  b. Setup a program with two levels of intensity. Set 50% as the first light intensity (equivalent to 1 W/cm) and length of exposure to 20 s.
    ***Note:*** Equipment settings for photoconversion should be optimized, other equipment can be used for this step i.e., SP500 spot UV curing (USHIO). Alternate equipment would require further validation.
  c. The second step should be set to 0% intensity and 5 s of exposure. Set the program to undergo nine cycles between 50% and 0% light intensity.
14. Prepare mice for photolabeling.
  a. Anesthetize mice with 5% isoflurane in an induction chamber.
  b. Transfer to a face mask and reduce the isoflurane to 2–3% and monitor the mouse to ensure stable anesthesia by checking breathing rate and pedal reflex.
15. Tumor photoconversion.
  **CRITICAL:** Near-UV light is harmful, wear UV protective glasses to prevent any damage to the users’ eyes.
  a. To prevent tissue adjacent to the tumor from being photoconverted, use a piece of light impermeable material with a hole in the center to shield nearby areas ensuring only the tumor will be exposed to near-UV light and photoconverted.
    ***Note:*** The shielding can be made of any light impermeable material, a wooden block was use here (See Figure 2).

### Generating single cell suspension from tissues

**Timing: 1–2 h**

17. Isolating cells from spleen.
  a. Dissect out the spleen, then carefully remove any fat attached and place into a 1.5 mL Eppendorf tube with 1 mL RPMI.
  b. Place a 70 μm cell strainer over a 50 mL flacon tube, add the spleen, and manual press the spleen through the strainer using the plunger from a 5 mL syringe.
  c. Thoroughly wash the strainer with 10 mL FACS buffer then centrifuge the falcon tube at 400 × *g* for 5 min at 4°C then discard the supernatant.
  d. Using a P100 pipette, resuspend the spleen in 1 mL FACS buffer until the suspension is homogenous, then add 9 mL of FACS buffer and pass through a 70 μm cell strainer into a 50 mL flacon tube.
  e. Centrifuge the falcon tube at 400 × *g* for 5 min at 4°C and discard the supernatant.
  f. Resuspend the pellet in 1 mL Gey’s solution and incubate on ice for 5 min.
  g. Centrifuge the falcon tube at 400 × *g* for 5 min at 4°C and discard the supernatant.
  h. Resuspend the cell pellet in 2 mL FACS Buffer and keep on ice ready for use.
18. Isolating cells from tumors.
  a. Dissect out the tumor and place into a 1.5 mL Eppendorf tube in 1 mL RPMI.
  b. Transfer tumor into a 1.5 mL Eppendorf with 100 μL RPMI and cut up tumor into 1–2 mm pieces using scissors.
    ***Note:*** Tumors are expected to be 100–200 mg in weight, larger tumors may need to be split in half to be processed before recombining the digested suspension.
  c. Add 1 mL of enzyme digestion mix, close the Eppendorf and invert to mix the sample before incubating on a shaking heat block set to 37°C and 1000 rpm for 25 min.
  d. Place a 70 μm cell strainer over a 50 mL flacon tube, add the tumor digestion mix, then manual press the tumor pieces through the strainer using the plunger from a 5 mL syringe.
  e. Centrifuge the falcon tube at 400 × *g* for 5 min at 4°C and discard the supernatant.
    ***Optional:*** If the cell pellet consists of aggregated clumps consider resuspending in FACS buffer and filter through a 70 μm cell strainer for a second time to further remove tumor cell aggregates.
  f. Resuspend the cell pellet in 2 mL FACS Buffer and keep on ice ready for use.
19. Isolating cells from lymph nodes.
  a. Dissect out the lymph node and place into a 1.5 mL Eppendorf with 200 μL RPMI.
  b. Using scissors, cut up the lymph not so no large visible pieces remain.
    ***Optional:*** Digestion of lymph nodes is not required to isolate most cell types but is advised to improve recovery of dendritic cells.
  c. Add 1 mL of enzyme digestion mix, close the Eppendorf and invert to mix the sample before incubating on a shaking heat block set to 37°C and 1000 rpm for 25 min.
  d. Place a 70 μm cell strainer over a 50 mL flacon tube, add the lymph node digestion mix, then manual press any pieces of lymph node through the strainer using the plunger from a 5 mL syringe.
  e. Centrifuge the falcon tube at 400 × *g* for 5 min at 4°C and discard the supernatant.
  f. Resuspend the cell pellet in 2 mL FACS Buffer and keep on ice ready for use.
    ***Optional:*** Cells can be restimulated *ex vivo* to assess production of intracellular proteins i.e., cytokines and granzymes.

### Staining for flow cytometric analysis

**Timing: 2–4 h**

20. Plate samples into a 96-well plate and centrifuge at 400 × *g* for 3 min, before decanting the supernatant.
21. Incubate all samples with 100 μL FACS buffer containing anti-CD16/32 Fc blocking antibodies for 20 min at 4°C.
22. Centrifuge at 400 × *g* for 3 min and discard the supernatant.
23. Gently resuspend cell pellets in 100 μL of prepared antibody staining mix (see Table 1) and incubate for 30 min at 4°C.

**Table 1:** Staining details for different target proteins

Antigen/stain	Fluorophore	Dilution
LIVE/DEAD Viability stain	Near IR 775	1 in 1000
CD16/32	Unconjugated	1 in 200
CD45	BV605	1 in 300
CD3	BUV737	1 in 200
CD4	BV786	1 in 200
CD8	PE-Cy7	1 in 200
CD11c	PE-Dazzle594	1 in 300
B220	BUV395	1 in 200
Foxp3	eFluor 450	1 in 100
LY-6C	BV711	1 in 300
LY-6G	BV650	1 in 300
F4/80	eFluor 660	1 in 200
MHCII	BV510	1 in 400

***Note:*** Include single color controls in addition to Kaede green, and Kaede red. Kaede red single-color controls can be obtained by photoconverting isolated splenocytes with the Dymax BlueWave QX4 system as before.

24. Centrifuge at 400 × *g* for 3 min and discard the supernatant, then wash cells in PBS before centrifuging again.
25. Resuspend cell pellets in 100 μL of PBS containing LIVE/DEAD viability dye and incubate for 15 min at 4°C.
26. Centrifuge at 400 × *g* for 3 min and discard the supernatant.

**Pause point:** Both green and red variants of the Kaede protein can be fixed using BD Cytofix without a significant loss of signal. Resuspend fully stained cell pellets in 100 μL BD Cytofix and incubate for 30 min at 4°C. Store fixed cells at 4°C covered in foil in the dark until use, ideally within 2–3 days.

***Optional:*** Fixed cells can be permeabilized with either BD Cytoperm or Foxp3 transcription factor permeabilizing buffer to stain for intracellular targets such as cytokines or transcription factors. Follow manufacturer’s guidelines.

27. Resuspend cells in 300 μL FACS buffer, add counting beads if required, and acquire samples on a flow cytometer.

## Expected outcomes

**Figure 3:**
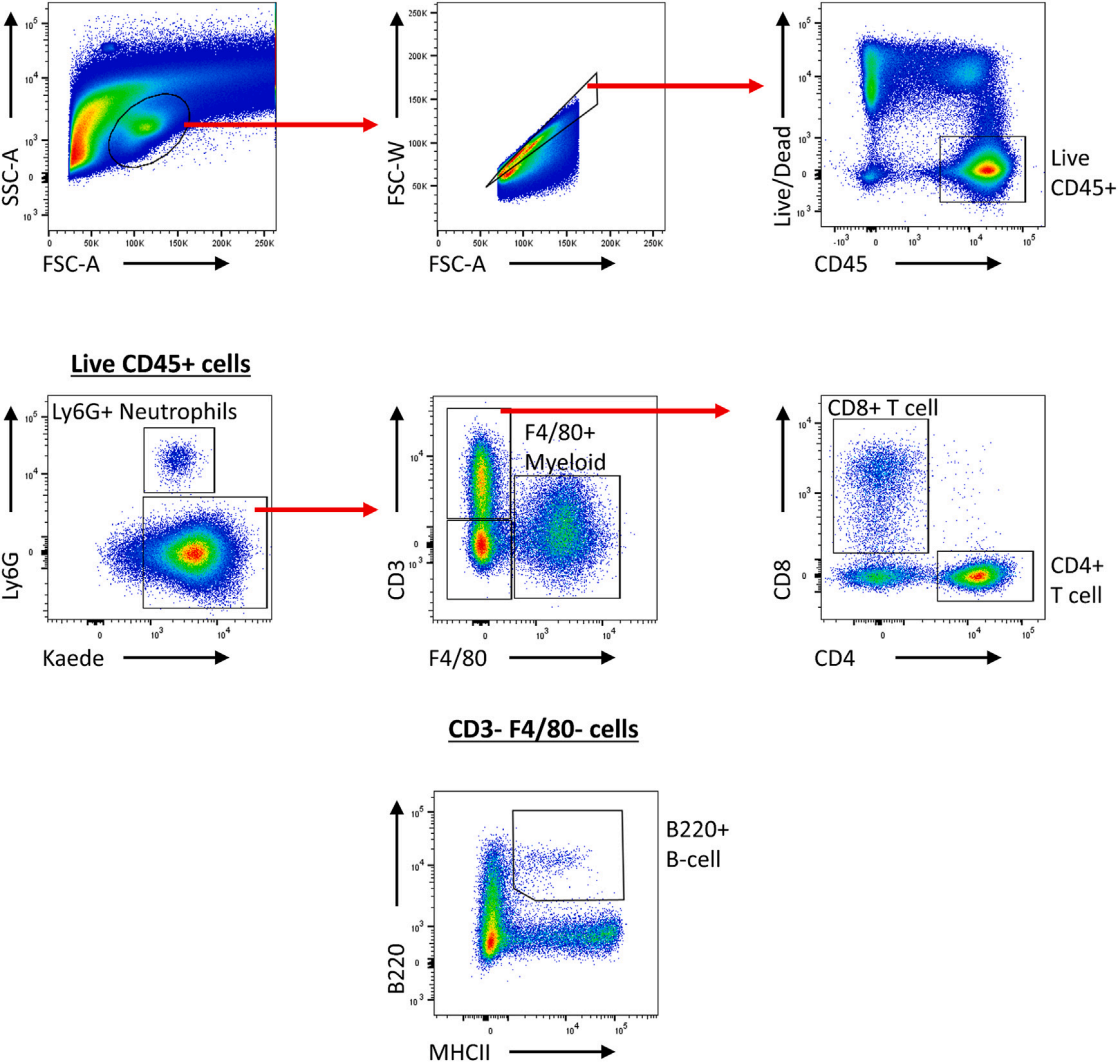
Gating strategy for intratumoral immune subsets Representative flow cytometry gating for immune subsets (T cell, B cells, and myeloid cells) within tumors.

**Figure 4:**
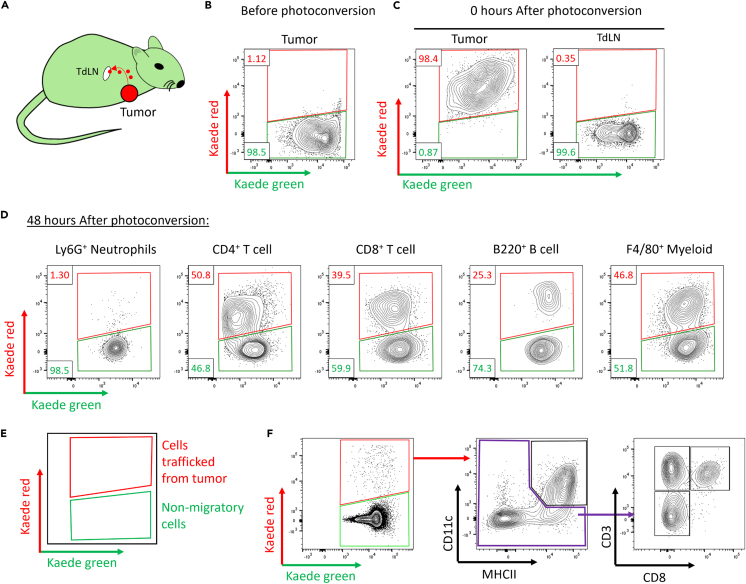
Tracking intratumoral immune cell dynamics and egress to lymph nodes using temporal labeling of the tumor immune compartment Cartoon depicting transcutaneous labeling of an m.f.p implanted E0771 tumor (A). Representative flow cytometry plots showing Kaede red and kaede green expression by CD45+ cells isolated from E0771 tumors before photoconversion (B), and site-specific temporal labeling immediately after photoconversion (C). Representative flow cytometry plots showing Kaede red and Kaede green expression by E0771 tumor infiltrating immune cells 48 h after tumor photoconversion (D). Illustration depicting a flow cytometry plot showing migration of Kaede red^+^ cells into the TdLN (E). Representative flow cytometry plot of migratory Kaede red^+^ cells in the TdLN, demonstrating gating for CD11c^+^ MHCII^+^ DC, CD3^+^ CD8^+^ T cells, CD3^+^ CD8^-^ T cells, and CD11c^-^ MHCII^-^ CD3^-^ ‘other’ cells (F).

Using simple canonical markers of immune cells, users should be able to identify an array of immune cell types, including neutrophils, T cell subsets, and the myeloid compartment within tumors (Figure 3). We expect to see ∼99% of non-tumor cells to be Kaede green^+^ in any given tissue. Immediately after photolabeling, all cells within the tumor should be Kaede red^+^ without photolabeling cells in the nearby lymph node as presented in Figures 4A–4C. The proportion of Kaede red cells within tumors will decrease over the following 0–48 h post photoconversion as newly entering Kaede green^+^ cells infiltrate the tissue (Figure 4D). This is a continuous process and occurs at varying rates for different immune cells. Similarly, this protocol enables tracking of dendritic cells which were within tumors at the time instance of labeling but have since migrated into draining lymph nodes (Figure 4E). In addition, other cell types can be tracked into the secondary lymphatics. This approach can be further dissected looking at multiple time points post labeling to map out the temporal dynamics of cellular migration in both s.c. and m.f.p engrafted tumors.

## Limitations

This protocol is designed to label small tumors ∼6 mm^2^ in the initial stages of tumor development, that form in easily accessible anatomical locations. Photolabeling of tumors in other locations may be possible but labeling efficiency heavily relies on the ability to expose the target tissue to light, therefore orthotopic tumors, for example, in the lung or liver would not be possible to label using transcutaneous light exposure. In addition, this approach relies on relatively short time frames <1 week to ensure detection of the Kaede red form which can be diluted by cellular proliferation or immune cell survival, or native protein turnover within cells.

## Troubleshooting

### Problem 1

Poor photoconversion efficiency (see step 13–15).

### Potential solution

**Figure 5:**
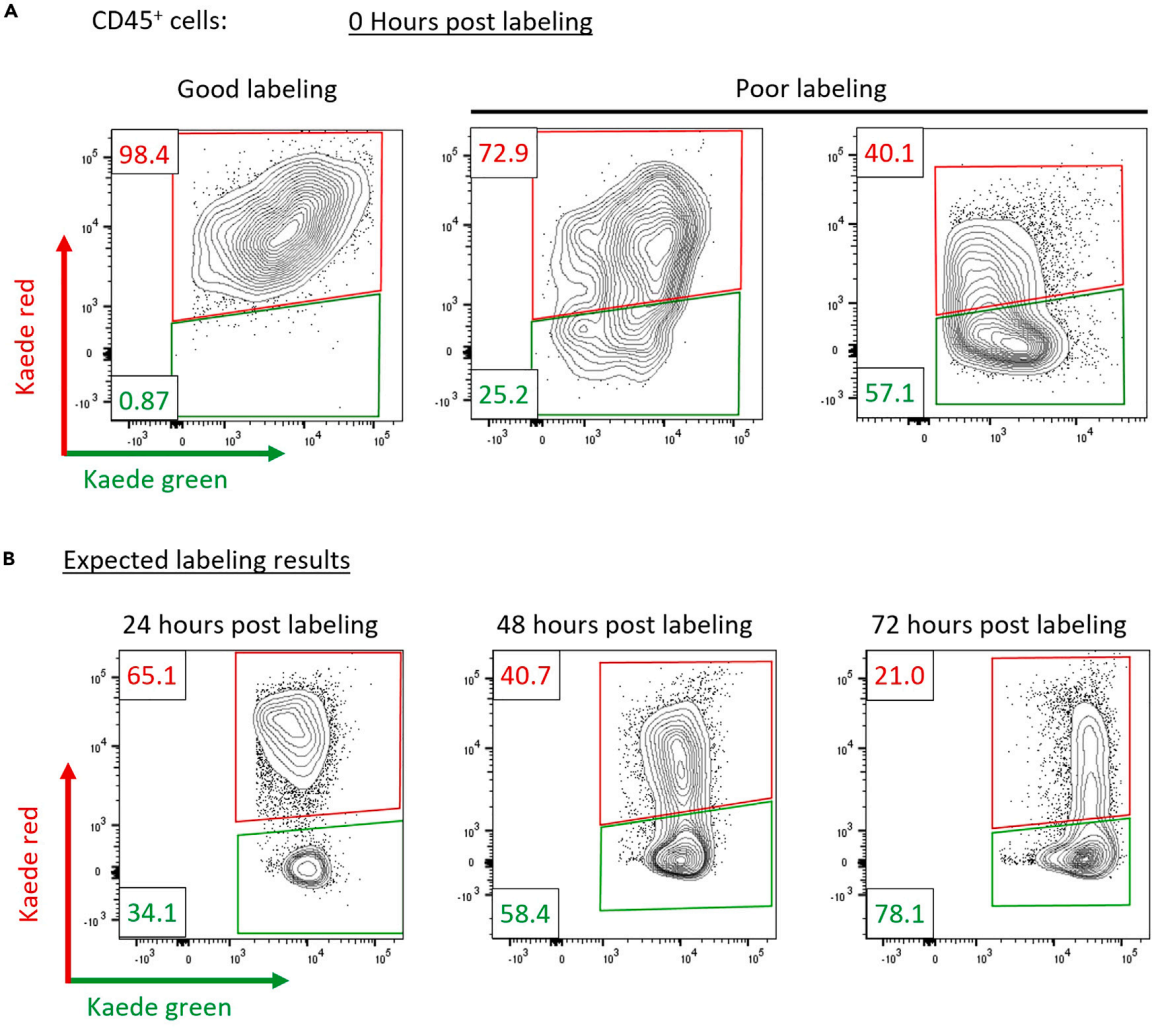
Incomplete photoconversion of Kaede Representative flow cytometry plots showing Kaede red and green expression immediately after photoconverting E0771 tumors depicting both complete and poor photolabeling efficiency shown (left and right respectively) (A). Representative flow cytometry plots of CD45^+^ cells showing Kaede red expression 24, 48, and 72 h after photolabeling the tumor (B).

Tumor photoconversion efficiency varies between mice due to differences in tumor size, shape, and the application of near-UV light. Assuming tumors are similar, photoconversion efficiency can be improved by optimizing the intensity of near-UV light, length of exposure, and distance from the light source to tissue. We recommend ensuring the entire tumor is exposed to light during photoconversion, then either increasing light intensity at 2% intervals, or increasing the length of exposure in each cycle by a second until photoconversion is achieved (Figure 5A).

During the photoconversion procedure, it is common to see the targeted tissue glow in a manner suggestive of some diffuse reflection of light within the tissue. Whilst not factually informative, from experience, this visual indication is suggestive of an ideal distance between the light source and tissue, and therefore photoconversion efficiency. Immediately after photoconversion, the skin above the tumor should appear red in color, indicating the presence of the Kaede red protein within the skin, and efficient photoconversion.

### Problem 2

Kaede green and Kaede red Compensation.

### Potential solution

Using FITC or PE as alternatives to Kaede green and Kaede red generates inaccurate compensation values. Wherever possible use Kaede expressing cells isolated from non-photoconverted tissue for the Kaede green single-color control, and then photoconvert an additional aliquot of Kaede green single color control *ex vivo* to obtain a true Kaede red single-color control. WT or non-pigmented cells should be used for unstained controls, and to add a negative population in Kaede single color controls. If the Kaede green vs. Kaede red signal appears to tail either under or over compensated, ensure Kaede single color control is brighter than experimental samples. We have observed lower Kaede expression in lymphoid tissues (spleen and peripheral lymph nodes) and is therefore quite common when using splenocytes for the Kaede green single color. In addition, ensure the Kaede red single color has been completely photoconverted, losing all Kaede green signal.

### Problem 3

Loss of Kaede red signal.

### Potential solution

Although Kaede in both green and red forms is stable, Kaede red signal can be reduced by loss of the Kaede red protein due to natural turnover overtime, or by proliferation and dilution of the Kaede red protein. In models presented here, even in non-proliferating cells, we observe a reduction in Kaede red signal in intratumoral CD8^+^ T cells over the course of 5 days (Figure 5B). Therefore, it is advised to ensure the time point of analysis post photoconversion is appropriate given the decline of Kaede red signal overtime. In our hands we recommend looking within 5 days, although 7 days is possible assuming high photolabeling efficiency.

## Resource availability

### Lead contact

Further information and requests for resources and reagents should be directed to and will be fulfilled by the lead contact, David Withers (d.withers@bham.ac.uk).

### Technical contact

Further information and technical help should be directed and fulfilled by the technical contact, Isaac Dean (isaac.dean@icr.ac.uk).

### Materials availability

C57BL/6 Kaede mice (#RBRC05737) are available under MTA from RIKEN BRC. Other reagents and equipment in the study are readily available from the key resources table.

### Data and code availability

All data from the studies are present here, in Li et al.^1^ and/or Molostvov et al*.*^2^
